# Ultrasound‐Responsive Engineered Bacteria for Targeted Cancer Therapy: Strategies, Mechanisms, and Applications

**DOI:** 10.1002/advs.202523952

**Published:** 2026-03-01

**Authors:** Xueyao Wang, Wenjing Xu, Yafang Lu, Huaibin Yu, Zhengbao Zha, Xijun Zhang

**Affiliations:** ^1^ Department of Ultrasound Zhengzhou University People's Hospital Henan Provincial People's Hospital Zhengzhou P. R. China; ^2^ School of Medicine and Health Zhengzhou Research Institute, Harbin Institute of Technology Zhengzhou P. R. China; ^3^ School of Medicine and Health Harbin Institute of Technology Harbin P. R. China; ^4^ School of Food and Biological Engineering Hefei University of Technology Hefei P. R. China

**Keywords:** cancer therapy, engineered bacteria, nanocarriers, precision medicine, tumor targeting, ultrasound

## Abstract

In recent years, with the rapid advancement of synthetic biology and nanoscience, microbial therapy based on engineered bacteria has garnered renewed widespread attention and achieved remarkable research progress. Specifically, through biological and physicochemical methods, bacteria from diverse sources with unique characteristics are functionally modified and engineered to combine with conventional therapeutic approaches. This aims to overcome or mitigate the limitations of standard treatments in clinical applications. On this basis, the integration of exogenously responsive regulatory factors (e.g., ultrasound) with microbial therapy undoubtedly provides a more intelligent, precise, and promising therapeutic strategy for tumor treatment. In this review, we summarize the core methods for direct modification, mechanisms of action, and main application strategies of engineered bacteria in tumor therapy. Meanwhile, we focus on elaborating the forms and interaction mechanisms of combining ultrasound with engineered bacteria, and discuss in detail the latest research advances in their combined application for tumor therapy. A comprehensive understanding of their relationships and interaction mechanisms will provide theoretical support for the development of ultrasound‐regulated bioactive molecules and ultrasound‐responsive intelligent drug delivery systems, thereby further advancing the applications of ultrasound technology and microbial therapy in the biomedical field.

## Graphical abstract

This review elucidates URB design strategies, including genetic and physicochemical modifications, while dissecting the synergistic mechanisms governing spatiotemporal control. It further evaluates applications in targeted drug delivery, gene modulation, and immunotherapy, finally offering perspectives on overcoming translational barriers for clinical implementation.

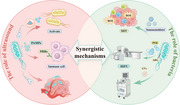

## 1 Introduction

Cancer continues to be a formidable challenge to global health, with its significant heterogeneity, drug resistance, and immunosuppressive microenvironment posing major challenges to treatment [1]. In recent years, bacterial therapies, with their unique tumor‐targeting and colonization abilities and multi‐dimensional anti‐tumor mechanisms, provide new ideas for cancer treatment [2]. Anaerobic bacteria may be preferentially accumulated in the tumor microenvironment (TME) through chemotaxis or hypoxia‐selective mechanism, or compete for nutrition with tumor cells, followed by release of toxins or immune regulation in situ, resulting in suppression of tumor growth and lower damage to normal tissues [3]. However, wild‐type strains suffer two challenges: highly pathogenic strains (e.g., Salmonella typhimurium AR1) are associated with host immunosuppression, structural and functional impairment of non‐target cells, and an augmented safety risk profile [4], while low‐pathogenic strains (e.g., Escherichia coli) lack tumorigenic activity [5]. In addition, the physical barrier of solid tumors in deep tissues hinders bacterial penetration, further limiting their clinical translation potential [6]. These limitations have spurred the addition of engineering philosophy to microbial function, in relation to the field of synthetic biology. Most of the engineered bacteria use attenuated bacteria [5, 7], which are engineered to have therapeutic potentials far beyond direct tumor lysis, including enzyme‐mediated prodrug conversion [8], gene therapy [9], and drug delivery [10], constructing a multifunctional platform to remodel the TME.

ontext, the convergence of cross‐disciplinary technologies has given rise to ultrasound‐responsive bacteria (URBs) therapy, which represents a major advance in precision control. Ultrasound technology offers a novel approach to precisely regulate bacterial therapy due to its non‐invasive nature, deep tissue penetration, and spatial and temporal controllability [11]. Low‐temperature‐sensitive liposomes (LTSL) loaded with drugs are attached to the bacterial surface. High‐intensity focused ultrasound (HIFU) triggers a phase transition in the LTSL through thermal energy, enabling precise regulation of drug release [12, 13]. Subsequent research has focused on the “heat‐sensitive switch” triggered by focused ultrasound (FUS), pioneering various new control methods and continuously optimizing them. For example, phase‐change ultrasound contrast agents can serve as nanocarriers for drug delivery, rupturing upon HIFU exposure to release the drug [14]. Designing temperature‐sensitive gene switches is another regulatory approach based on the targeted heating capabilities of ultrasound, achieving precise spatiotemporal induction of therapeutic transgene expression. When bacteria are exposed to ultrasound‐induced heat, they secrete antibodies, immune factors, apoptotic proteins, and immune checkpoint inhibitors in vitro, such as Interferon ‐γ [15], αCTLA‐4 [16], and azurin [17], thereby activating local immunity and avoiding systemic immune side effects. Notably, Abedi et al. further utilized Bxb1 integrase to achieve continuous release of immune antibodies after bacterial activation, which can accurately combat tumor cells to mitigate the risk of disease recurrence and reduces the frequency of clinical administration, thereby alleviating the treatment burden on patients [16]. With the development of URB technology, the synergistic design of nano‐sonosensitizers loaded with bacteria has significantly enhanced bacterial targeting and local reactive oxygen species (ROS) generation capacity, achieving effective integration of sonodynamic therapy (SDT) with bacteriotherapy [10]. Building on this, the use of ROS‐responsive covalent bonds (e.g., thioketal (TK) bonds, disulfide bonds, etc.), the delivery efficiency of chemotherapeutic drugs can be further improved to achieve spatiotemporally controlled release and pharmacokinetic optimization [18].

This review summarizes recent advances in the use of ultrasound‐responsive bacteria for cancer therapy. By examining four key aspects, it provides a comprehensive overview of URBs' developments: (1) The strategies of design and modification of URBs; (2) Molecular mechanisms underlying the interrelationship between ultrasound and bacteria; (3) The main application directions of URBs in cancer therapy; and (4) The challenges and future translational prospects of URBs. We hope this summary offers a theoretical framework and research directions to support the ongoing development of this pioneering therapeutic strategy, as well as practical guidance for its clinical implementation.

## 2 Design and Modification

As a cutting‐edge research focus in modern biochemical technology, the molecular design and functional modification of engineered bacteria have become central topics in tumor therapy. The primary bacterial species used can be categorized into two main groups: anaerobes and probiotics [19, 20]. Currently, the most studied ultrasound‐responsive tumor‐targeted live bacteria are non‐pathogenic *E. coli* [21], *B. longum* [22], and *S. typhimurium* [17]. These bacteria can be genetically recombined and precisely edited to confer ultrasound‐responsive properties at the microbial genome level, significantly enhancing their efficacy in tumor therapy [16, 17]. In addition, screened and modified bacteria can be effectively combined with ultrasound‐responsive materials [23]. Commonly used *E. coli* including DH5α [24], BL21 [14], Nissle1917 [25], offer advantages such as ease of genetic engineering modification and rapid growth. Bifidobacteria, as probiotics with high biological safety, can promote collagen deposition and increase tissue hardness without genetic modification, inhibit neovascularization, thereby continuously altering the acoustic environment of tumors, improving HIFU ablation efficiency, and extending the effective treatment window [26]. Salmonella exhibits natural toxicity against tumor cells, and the attenuated strain VNP20009, which has been clinically validated for safety, has become a prominent focus in genetic engineering bacterial therapy research [27]. By analyzing the characteristics of different bacterial species and comparing their therapeutic effects, researchers can select appropriate bacterial species according to specific needs and perform corresponding modifications and optimizations to achieve improved therapeutic outcomes. Currently, the construction of such engineered bacteria primarily involves genetic engineering techniques combined with nanotechnology to achieve biological and chemical modifications.

### 2.1 Biomodification

**FIGURE 1:**
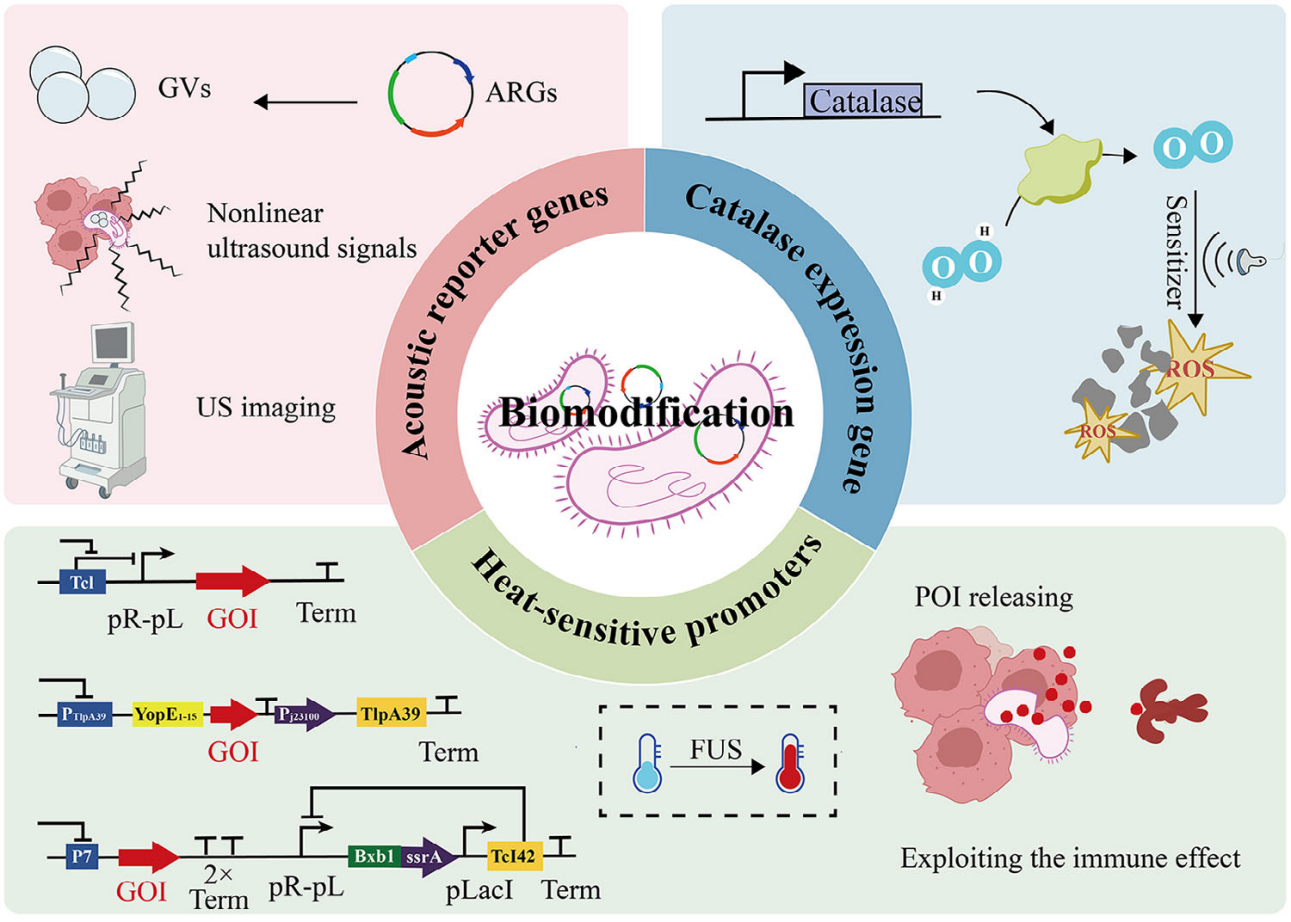
Biomodification strategies of ultrasound‐responsive bacteria. Reproduced with permission. [15] Copyright 2022, Springer Nature. Reproduced with permission. [17] Copyright 2024, Elsevier. Reproduced with permission [16]. Copyright 2022, Springer Nature.

Advancements in building and improving bacterial treatments have been contributed by new acoustic reporter genes, catalase, and heat‐sensitive expression genes (Figure 1). Ultrasound‐controlled targeting and treatment of bacteria have been achieved by genetic engineering and synthetic biology, which utilizes precision to manipulate bacterial genes to create a specific response to ultrasound.

The design of acoustic reporter genes (ARGs) has provided a high potential in real‐time tracking of bacterial behavior in natural in vivo systems in the bacterial biomodification field. In 2018, ARG1 and ARG2, developed by Shapiro and others, were shown to be expressed in *E. coli* and *S. typhimurium*. The vesicles of these ARGs are sound reflectors and allow gene‐encoded imaging using ultrasound. Gas Vesicles (GVs) produced can only offer linear ultrasonic contrast with a weak signal, and it is difficult to differentiate them from background tissue [28]. To address this, optimized ARGs with better nonlinear contrast and stability have been developed [29], which allow monitoring the presence of therapeutic microorganisms. This advancement will allow monitoring the presence of therapeutic microorganisms in the tumor in vivo and provide an opportunity to view the efficacy of the tumor and perform surgery with the help of ultrasound. ARG should be enhanced with optimization of the gene cluster expression kinetics and simplification of the structure in the future.

In addition, the expression of catalase by genetically engineered bacteria can overcome the limitations of the hypoxic TME on acoustic kinetic therapy [30]. Since most sonosensitizers require O_2_ as a substrate to generate ROS under ultrasound excitation, enabling bacteria to express catalase allows continuous catalysis of H_2_O_2_ to O_2_ within tumors. This approach not only alleviates the TME driven by hypoxia and improves the immunosuppression of tumors due to lactic acid stagnation, but also significantly improves the efficiency of SDT by inhibiting the expression of HIF‐1α, which offers a novel approach to acoustic immunotherapy [31].

The application of bacterial bioengineering combined with ultrasound goes far beyond this, with an increasing number of researchers developing sound‐controlled gene circuits. The core components are temperature‐sensitive transcriptional regulatory elements, such as TlpA [17, 32], and TcI [33]. By inserting these temperature‐sensitive elements into the λpL/pR [15] or PTlpA promoter [17], a “thermosensitive gate” is formed, ensuring complete silencing at 37°C. Ultrasound‐induced transient heating lifts this inhibition, triggering the expression of downstream therapeutic genes. Among these, TlpA39 dissociates from the PTlpA promoter when the temperature rises to 39°C. The switchover occurs within the temperature range triggered by low intensity pulsed ultrasound conventional diagnostic‐level ultrasound (0.4–0.5 W/cm^2^), significantly reducing the risk of thermal damage to deeper tissues [17]. Furthermore, by placing the serine integrase Bxb1 downstream of the temperature‐sensitive element, the attB/attP‐flanked therapeutic gene cassette can be flipped, transitioning the bacteria from a “silent” to a “continuous production” state. A single ultrasound application can achieve long‐term stable therapeutic gene expression [16]. Currently, genetically modified bacteria are typically combined with immunotherapy to mitigate the common systemic side effects associated with immunotherapy.

### 2.2 Physical and Chemical Reformation

**FIGURE 2:**
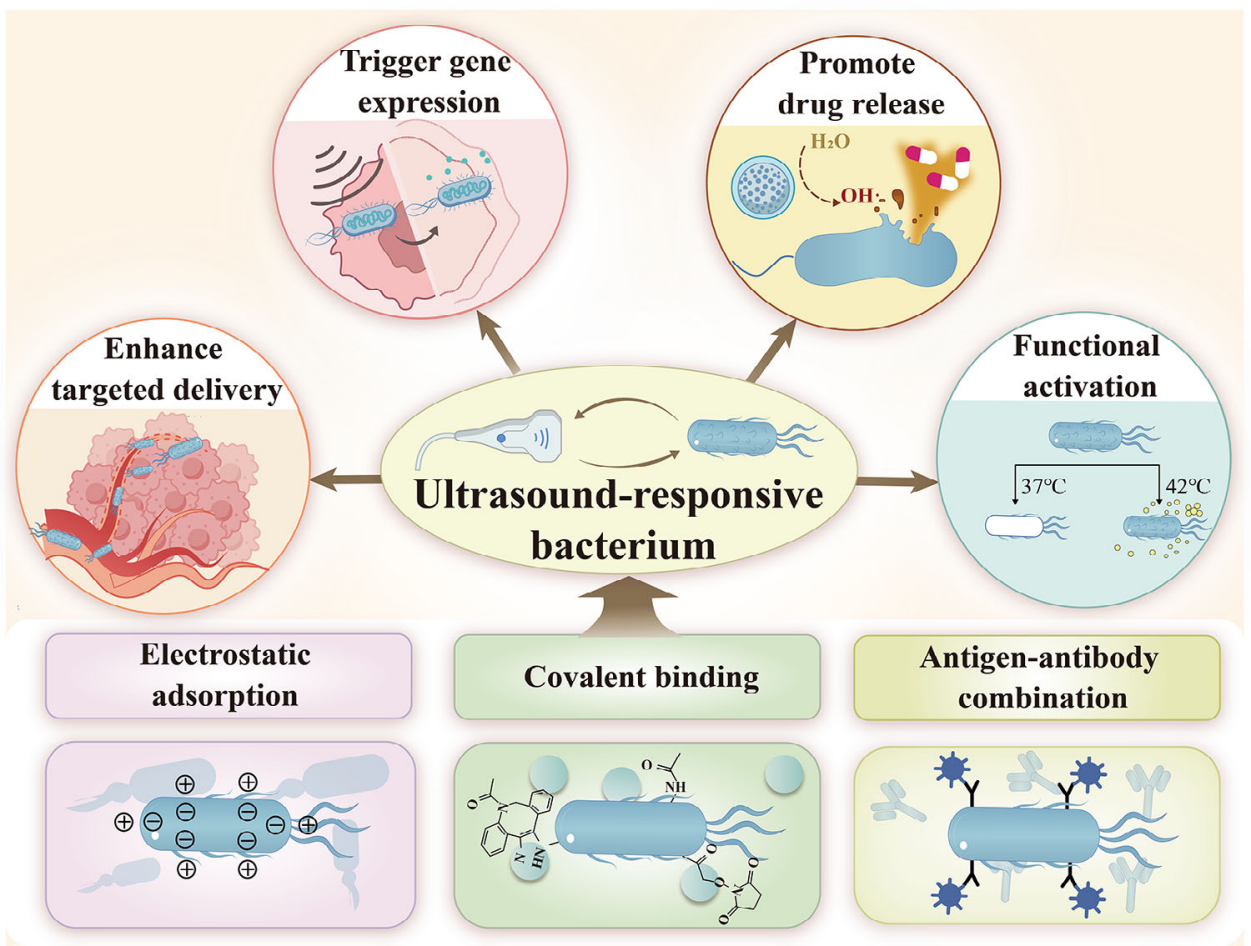
Chemical modification strategies of ultrasound‐responsive bacteria and their associated functions. Reproduced with permission [10]. Copyright 2024, Wiley‐VCH GmbH.

The combination of bacteria with ultrasound‐responsive materials has led to an innovative strategy for cancer treatment, demonstrating excellent biocompatibility and biosafety. Currently developed ultrasound‐responsive nanocarriers not only maintain bacterial targeting to tumor tissues, but also enable controlled drug release upon ultrasound stimulation, significantly improving therapeutic efficacy [34]. These systems typically utilize biocompatible materials such as poly (lactic‐co‐glycolic acid) (PLGA) and liposomes as carriers to ensure safety while integrating multiple functions. The main binding strategies commonly employed include electrostatic adsorption, covalent modification, and antigen‐antibody binding (Figure 2).

Electrostatic adsorption is a simple and efficient method for bacteria‐material binding [10]. Bacteria with negatively charged surfaces are able to stably bind to drug‐carrying cation‐modified PFH/PLGA nanoparticles via electrostatic adsorption. The application of this binding system results in a significant expansion of coagulative necrosis in the treated region, decreases the HIFU ablation energy efficiency factor (EEF), and improve tumor ablation efficiency [35]. For example, Jiang et al. demonstrated that bifidobacteria could be effectively combined with cationic lipid nanoparticles (CL‐ICG‐PFH‐NPs) by electrostatic adsorption, forming an URB with synergistic biological targeting effects [36]. Wang et al. utilized polyethyleneimine (PEI)‐modified PLGA nanoparticles to achieve the optimization of the potential reversal and positive charge density. By utilizing the electrostatic adsorption of bifidobacteria, a functionalized composite system of PEI‐PLGA‐NaHCO_3_‐NPs and tumor‐homing bacteria was successfully constructed, further enhancing binding efficiency and stability [37].

To further enhance the stability of the bacteria‐material interaction, researchers introduced a covalent modification approach, wherein ultrasonic‐responsive materials were fixed to the surface of target bacteria via covalent coupling. Common covalent modification methods include alkylation, acylation, phosphorylation, methylation and amidation. This approach not only improves binding selectivity, but also significantly prolongs the circulation time of the composite system in vivo. Luo et al. combined PLGA nanoparticles (PFH/PLGA NPs) with *B. longum* by the carbodiimide method, which markedly enhanced breast cancer ablation under HIFU treatment [38]. Du et al. developed a composite system consisting of the sonosensitizers hematoporphyrin monomethyl ether (HMME) and perfluoropentane (PFP)‐loaded PLGA nanodroplets (HPNDs), which were covalently bound to the *E. coli* Nissle 1917 to form the HPNDs@EcN complex [39].

The antigen‐antibody binding mode is highly specific. Chen et al. successfully crosslinked biotinylated lipid nanoparticles (PFH/BL‐NPs) encapsulated with PFH and streptavidin‐conjugated antibodies against *B. longum* for the first time in vitro, which resulted in the binding of the two in a clustered manner, enhancing the targeting capability of the PFH/BL‐NPs for solid tumors and their retention time inside the tumors, thus providing better imaging and therapeutic effects, providing an effective means to construct complexes for tumor therapy [40].

### 2.3 Comparative Analysis and Synergistic Strategies

Although both biomodification and physicochemical reformation have significantly advanced the functionality of URBs, each strategy possesses distinct advantages and limitations. Genetic engineering allows for the intrinsic, heritable expression of functional proteins (e.g., ARGs and therapeutic enzymes) with precise controllability through gene circuits. However, this approach is often constrained by the bacteria's metabolic burden and stringent biosafety regulations [5, 16]. In contrast, physicochemical surface modification offers a versatile platform for loading a wide range of payloads—including chemotherapeutic drugs and nanomaterials—without altering the bacterial genome, though it faces challenges regarding payload dilution during cell division [6, 10].

To overcome these individual limitations, a cutting‐edge trend involves the synergistic integration of both strategies. This dual‐modification approach combines the biological precision of gene editing with the functional diversity of material loading. For instance, recent studies have successfully enhanced tumor ablation by combining genetically expressed GVs with chemically loaded phase‐change droplets [14], or achieved versatile theranostics by coating acoustic reporter bacteria with functional nanoparticles [35, 84]. These dual‐modified bacteria demonstrate that combining genetic and physicochemical engineering is a superior strategy for developing next‐generation intelligent URBs.

## 3 Fundamental Principles for Therapy

### 3.1 Mechanisms of Bacteria‐Mediated Therapy

**FIGURE 3:**
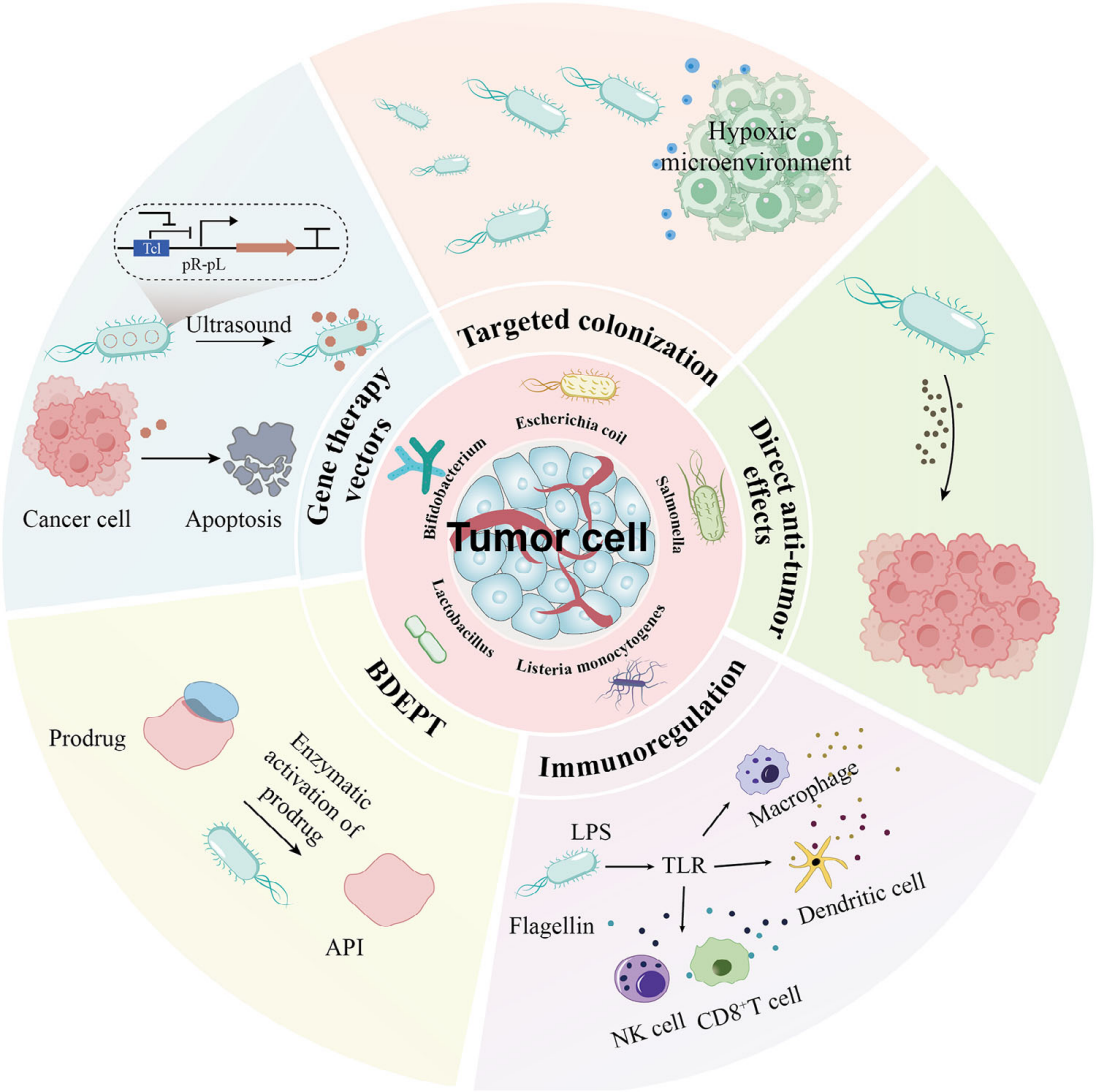
Schematic illustration of anti‐tumor effects of bacteria.

Bacterial therapy, an emerging strategy for tumor treatment, has received widespread attention in oncology in recent years [41]. Scientists have produced more effective and less toxic bacterial strains through recombinant DNA technology. Currently, the mechanisms of bacterial anti‐tumor activity are complex and diverse, mainly including the following aspects (Figure 3): (1) Bacterial targeting of the TME; (2) Direct anti‐tumor effects; (3) Immunomodulatory effects; (4) Enzyme‐catalyzed prodrug, and (5) Bacterial vector strategies in gene therapy. These mechanisms work synergistically to exert anti‐tumor effects.

#### 3.1.1 Targeted Colonization

One of the core advantages of bacteriotherapy is its ability to selectively localize and colonize tumor tissue [42]. The mechanisms by which bacteria target the TME are multifaceted. First, the rapid growth of tumor tissue often exceeds its vascularization rate, leading to the formation of hypoxic areas within the tumor [43]. Hypoxia and low pH levels promote significant tumor targeting by bacteria [44]. Second, tumor blood vessels have structural and functional abnormalities compared to normal vessels, with higher permeability and leakage [45], allowing bacteria to more easily leak out of blood vessels and enter tumor tissues [46]. Third, there are many chemokines and metabolites in the TME, which can be used as “navigation signals” for bacteria, guiding their migration to the tumor site [19]. Kasinskas et al. found that Salmonella mediates chemotactic movement via aspartate and serine receptors, while ribose/galactose receptors mediate bacterial migration into necrotic tumor regions [47]. Upon reaching the tumor, bacteria induce tumor cell apoptosis (e.g., UA‐mediated apoptosis), releasing cellular debris and nutrients (e.g., amino acids, and sugars), which further support bacterial proliferation in hypoxic regions [48]. Furthermore, immunosuppressive factors secreted by various cells within the TME not only inhibit anti‐tumor immunity but also reduce bacterial clearance [43]. In summary, the aforementioned multiple complementary mechanisms enable bacteria to efficiently target the TME and establish long‐term colonization at stable levels, offering a novel and powerful strategy for cancer therapy [49]. For instance, this approach enhances nanoparticle enrichment, improves molecular imaging and therapeutic efficacy, and extends the therapeutic window [36, 50].

#### 3.1.2 Direct Anti‐Tumor Effects

The direct antitumor effects of bacteria involve a variety of mechanisms, among which nutrient competition being a key component, in which bacteria inhibit the growth of tumor cells by competing for essential nutrients [51]. In addition, certain bacteria function as microbial agent factories, secreting cytotoxic substances that directly cause tumor cell death [10] or interfere with tumor cell glucose metabolism or lipid metabolism [52]. For example, proteins released by Clostridium species can trigger apoptosis in tumor cells [53], while toxins produced by Salmonella can induce cell death or autophagy, enhancing the bacteria's antitumor effects [54]. Furthermore, short‐chain fatty acids (SCFAs) and other bacterial metabolites can regulate the pH of the TME, thereby inhibiting tumor cell proliferation and invasion [55].

#### 3.1.3 Immunoregulation

Bacteria further activate immune pathways, which promotes immune system recognition and is essential for immune response. Bacterial components (e.g., flagellin, lipopolysaccharide, etc.) can activate the toll‐like receptor (TLR) signaling pathway, which induces and promoting the infiltration of a variety of immune cells (e.g., dendritic cells, macrophages, and neutrophils) into the tumors, and can also directly or indirectly stimulate the release of a large number of pro‐inflammatory cytokines from immune cells [56]. In addition, bacterial colonization can alter the TME, making it more susceptible to immune attack and enhancing the efficacy of other therapeutic approaches by inducing inflammation and modulating cytokine levels. Recent studies have found that bacteria exploit the IL‐10 receptor (IL‐10R) hysteresis phenomenon, causing immune cells in the TME to maintain elevated IL‐10R expression and respond more effectively to IL‐10 signaling. This phenomenon is widely observed across multiple immune cell types, including CD8^+^ T cells, tumor‐associated macrophages (TAMs), and tumor‐associated neutrophils (TANs) [57].

#### 3.1.4 BDEPT

Bacterial‐directed enzyme prodrug therapy (BDEPT) has the potential to be an effective therapeutic delivery system by exploiting the capability of bacteria that transform inactive prodrugs into active cytotoxic molecules in the tumor location. The two‐step process minimizes systemic toxicity and increases the local effects of medication [58]. Prodrugs such as glycyrrhetinic acid can be converted through engineered *E. coli* into active metabolites that have a devastating tumor growth inhibitory effect in preclinical models [59]. The other illustration is nitroreductase NTR of Clostridium, which transforms the prodrug, such as CB1954, into extremely toxic compounds that cause death to cancer cells and enhance treatment [60].

#### 3.1.5 Gene Therapy Vectors

Bacteria may also be used as delivery vehicles to administer genes that have been chemotherapeutic, e.g., antitumor genes, into tumor tissues with the help of vectors. Targeted antitumor effect: Tumor‐specific promoters used to target therapeutic genes largely work in tumor tissue [9]. It enhances the treatment of tumors. Genetic engines are capable of TNF or IFN expression by ultrasonic stimulation in genetically engineered bacteria. These cytokines have ability to destroy tumor cells or boost immune responses [15].

### 3.2 Mechanisms of Ultrasound‐Mediated Therapy

**FIGURE 4:**
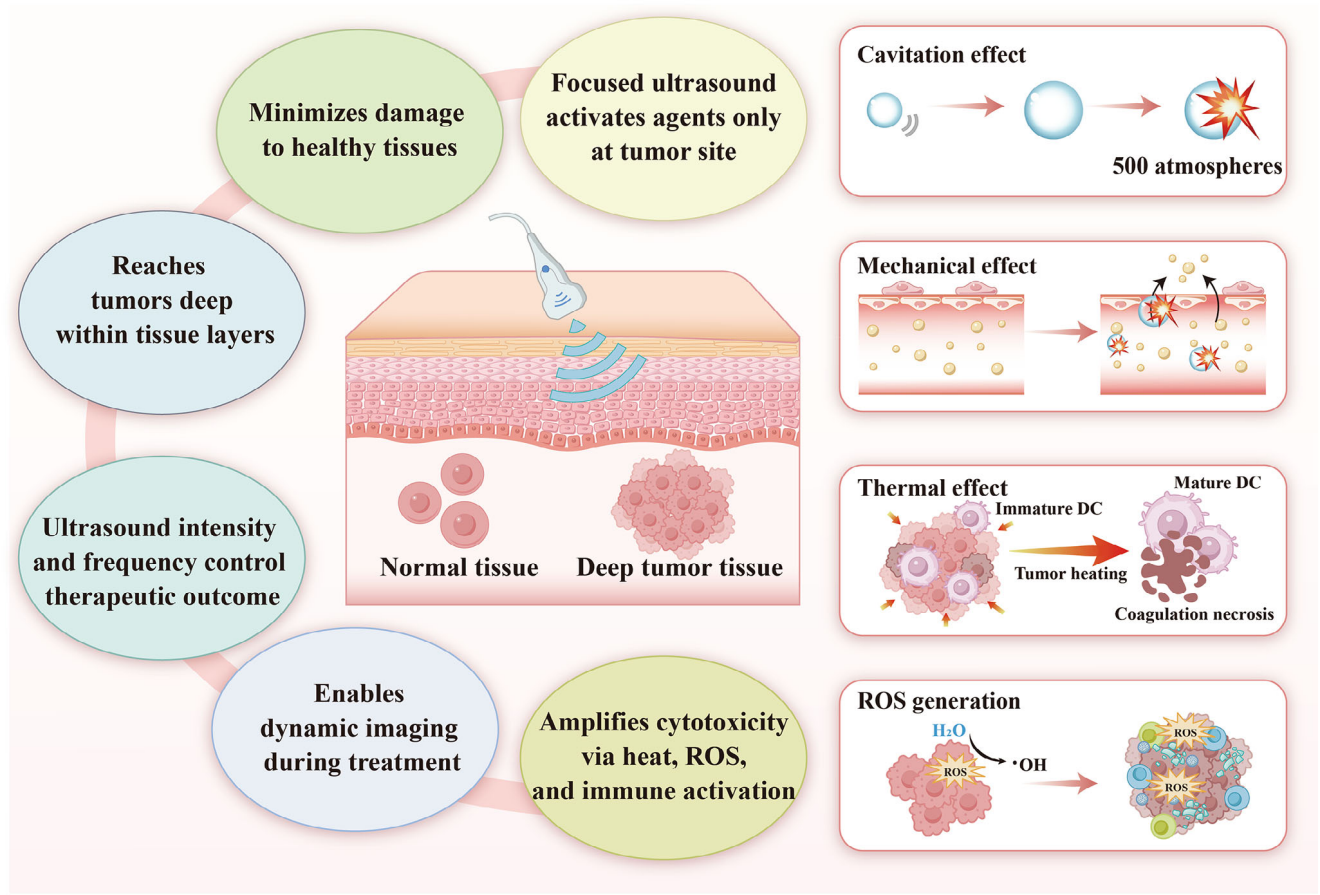
Mechanisms of action and advantages of ultrasound in the treatment of deep‐seated tumors.

As a non‐invasive means of physical stimulation, ultrasound has shown broad potential for application in gene expression and drug delivery in recent years [61]. Its unique spatiotemporal controllability makes it a promising tool for tumor therapy. The application of ultrasound in bacteriotherapy not only enables direct cell killing but also further improve the therapeutic efficacy by improving drug delivery and activating the immune response [62]. The four main effects of ultrasound—cavitation, mechanical, thermal, and ROS act synergistically in bacteriotherapy to enhance tumor cell killing (Figure 4).

#### 3.2.1 The Cavitation Effect

The cavitation effect is the predominant physical effect of ultrasonic therapy and has number of biological effects. When the pressure of the ultrasound‐induced fluctuations grows and breaks due to tissue microbubbles, cavitation takes place. The technique establishes a transient high temperature and pressure condition, killing tumor cells [63]. For instance, gas vesicles (GVs)—protein‐shelled nanostructures expressed by certain bacteria—can serve as endogenous, stable cavitation nuclei. These GVs significantly lower the acoustic energy threshold required to trigger cavitation and enhance the localized deposition of ultrasonic energy due to their unique acoustic scattering properties [28]. Alternatively, phase‐change agents such as PFH can be loaded onto bacteria. Upon ultrasound irradiation, PFH undergoes acoustic droplet vaporization (ADV), transitioning from a liquid nanodroplet to a gas microbubble. This phase transition generates substantial expansion stress and provides new nuclei for subsequent inertial cavitation, thereby amplifying the mechanical disruption within the tumor microenvironment [35].

#### 3.2.2 The Mechanical Effect

Most mechanical effects of ultrasound are due to the production of pressure changes due to the transmission of ultrasoniwaves, and additionally, the changes may elevate vascular permeability, reduce interstitial fluid pressure, and also enhance TME, which enhances better penetration to tumor tissue by bacteria [64]. Second, this difference may lead to sonoporation, which is the temporary formation of micropores in cell membranes [65]. This enhances the uptake of macromolecular therapeutic molecules such as chemotherapeutic drugs, antibodies, and fragments of genes into tumor cells, enhancing cell efficacy. In addition, Joiner et al. emphasized that ultrasound mechanical perturbation integrates immune cell activation by stimulating liberation of damage‐associated molecular patterns (DAMPs) and heat‐shock proteins (HSPs) to boost the antitumor immune reaction [66].

#### 3.2.3 The Thermal Effect

The thermal effect of ultrasound refers to the biological phenomenon caused by the absorption and conversion of acoustic energy into heat energy during its propagation through biological tissues. Within the TME, such ultrasound‐induced elevated temperatures not only cause thermal ablation and hyperthermia of tumor cells but also effectively promote vasodilation, increasing vascular permeability. This shift increases the delivery and distribution of the chemotherapeutics and nanomedicines to the tissues of the tumor [67, 68]. For example, Ektate and colleagues achieved precise drug release at tumor sites by combining LTSL with HIFU heating. In addition, it was found that this local thermotherapy could also shift pro‐tumor M2 macrophages into anti‐tumor M1 macrophages, achieving dual synergistic effects of chemotherapy and immune modulation [12]. Further, the synergistic ultrasound effect with mild hyperthermia favorably increases the tumor cell sensitivity to radiotherapy and certain DNA‐damaging chemotherapeutic agents by inhibiting the repair of DNA damage associated proteins, such as BRCA2, achieving increasingly effective therapeutic effects [69].

It is important to note that the thermal and mechanical effects of ultrasound are often intimately coupled rather than acting in isolation during actual treatment. For instance, the violent collapse of cavitation bubbles (mechanical effect) can generate localized high temperatures, thereby contributing to thermal ablation [70]. Conversely, hyperthermia induced by ultrasound absorption can alter tissue viscoelasticity, making tumor cells more susceptible to mechanical disruption. This synergistic interplay between thermal and mechanical mechanisms plays a critical role in enhancing the overall therapeutic efficacy of URBs.

#### 3.2.4 ROS Generation

ROS are a class of compounds derived from oxygen molecules, primarily including superoxide anion (·O_2_^−^), hydroxyl radical (·OH), and hydroperoxide (H_2_O_2_). The generation of ROS by ultrasound primarily relies on the cavitation effect [71]. Cavitation creates localized environments of extreme heat and pressure, with temperatures reaching thousands of kelvins and pressures exceeding hundreds of atmospheres. Under these extreme conditions, water molecules thermally decompose into ROS, such as hydroxyl radicals (·OH) [72]. These hydroxyl radicals can inflict oxidative damage on cells and tissues. Oxidative stress represents one of the primary mechanisms by which ROS affect cells; excessive ROS can damage intracellular lipids, proteins, and DNA, leading to cellular dysfunction and apoptosis. Significant interest has emerged in applying ultrasound‐generated ROS to tumor therapy, especially within SDT. It utilizes sound‐sensitive agents to generate increased ROS via ultrasonic irradiation in tumor cells, thereby further inducing immunogenic cell death [73].

### 3.3 Interaction Mechanisms of Bacteria and Ultrasound

**FIGURE 5:**
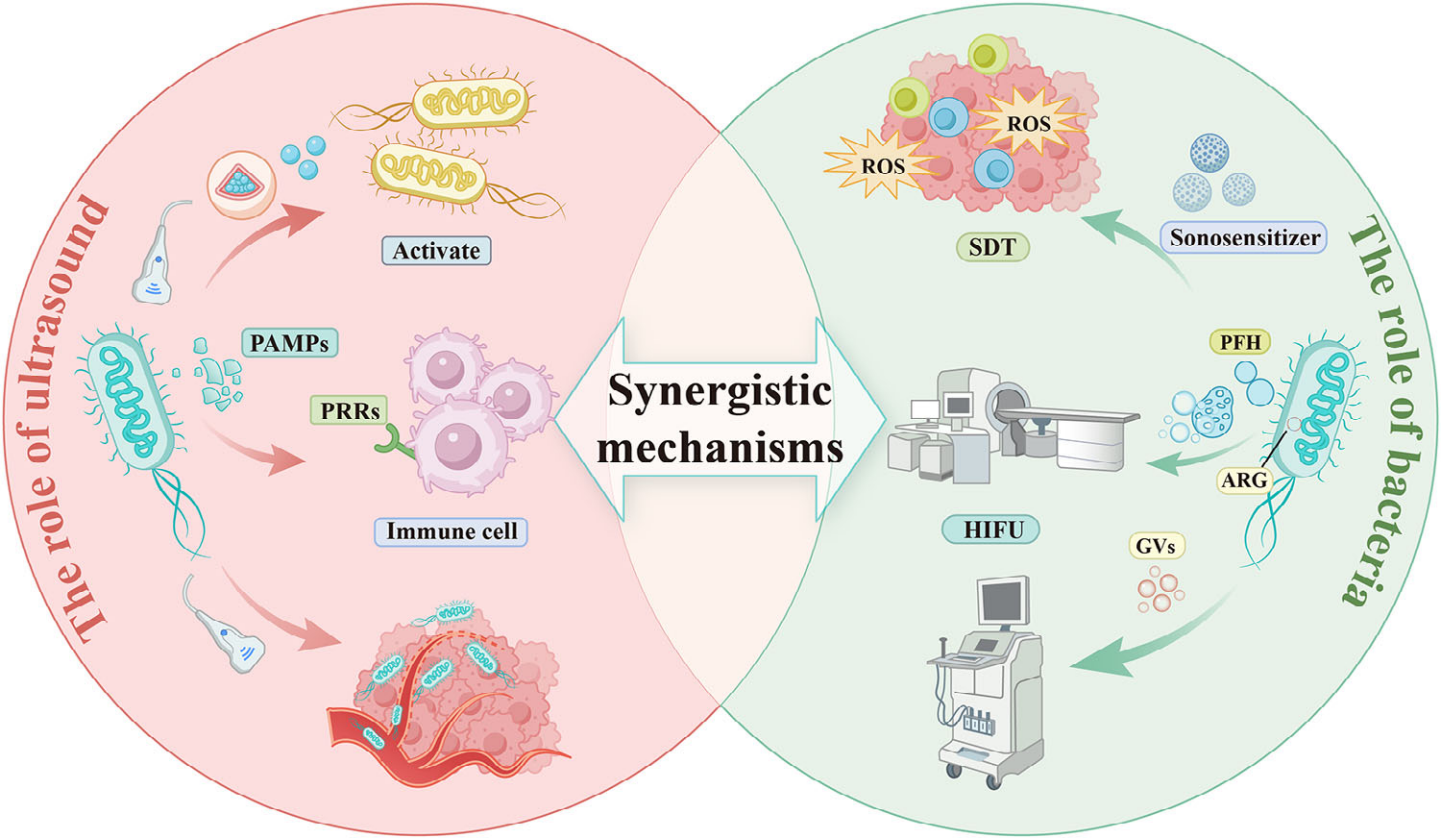
Schematic diagram of the mutual synergistic effect between ultrasound and bacteria, including the strategic mechanism of spatiotemporal controllable bacterial treatment by ultrasound, as well as the strategic mechanism of bacteria‐enhanced ultrasound treatment.

The application of ultrasound technology in bacterial therapy has increasingly attracted the attention of researchers. Currently, various therapeutic strategies utilizing ultrasound‐responsive bacteria have been developed, demonstrating excellent antitumor effects at tumor sites. In the following discussion, we briefly explore the underlying mechanisms, including how ultrasound endows bacteria with spatiotemporally controllable functions, enhances bacterial colonization, and amplifies ultrasound‐based therapy and imaging (Figure 5). This aims to provide insights and methodologies for subsequent researchers.

#### 3.3.1 Ultrasound‐Controlled Bacterial Therapy

In ultrasound responsive bacterial therapies, ultrasound can modulate temperature and mechanical stimulation spatiotemporally trigger bacterial carrier's function activation, for example, gene expression and drug release.

Ultrasonic waves induce temperature elevation through thermal effects, thereby activating gene expression in bacterial vectors equipped with thermosensitive genetic circuits (e.g., pBV220/cI857 or TlpA/TcI systems), as detailed in Section 2.1 [15, 16, 17, 74]. This mechanism enables precise spatiotemporal control of therapeutic protein production. In addition, the thermal effect of ultrasound can control the performance of temperature‐sensitive liposome and then realize drug release from bacterial carrier [14].

Furthermore, phase‐change agents like PFH can undergo a rapid liquid‐to‐gas transition under ultrasound, generating explosive expansion stress and localized microjets. These mechanical forces are sufficient to disrupt the structural integrity of nanocarriers, thereby liberating encapsulated drugs [35]. Finally, the expression of target gene is achieved by controlling the relationship between ROS responsive linkers and sonosensitizers used in Liang's group [18]. This remarkable cascade reaction strategy provides reference for further development of ultrasound responsive bacterial drug delivery system.

#### 3.3.2 Ultrasound‐Mediated Modulation of Bacterial Behavior

Recently, ultrasound has been shown not only to directly affect the behavior of bacteria but also to potentially influence bacterial colonization and spreading by modulating the TME. First, different parameters of ultrasound (including frequency, intensity, and pulse cycle) can affect bacterial growth and distribution. Low‐frequency ultrasound is more likely to induce cavitation effects, which can improve cell membrane permeability [64] and stimulate bacterial growth [75]. Second, FUS can enhance tumor vascular permeability within the TME through thermal ablation, thermal stress, and mechanical disruption. This disruption of the extracellular matrix further facilitates the infiltration of bacteria into tumor tissues [64, 66].

#### 3.3.3 Bacteria‐Enhanced Ultrasound Therapy

HIFU, as a new minimally invasive therapeutic technology, uses an acoustic transducer to precisely focus ultrasound waves emitted from outside the body on the target tissue, and with the help of the instantaneous generation of high temperatures (60‐100°C), induces coagulative necrosis of tumor cells [76, 77]. The thermal conversion efficiency is governed by the tissue's absorption coefficient and the focal intensity, which is fundamentally limited by acoustic attenuation (comprising absorption and scattering) during trans‐tissue propagation [78]. To enhance energy deposition within deep‐seated lesions, URBs can be engineered to modulate the acoustic impedance and cavitation threshold of TME [79]. Specifically, by URBs to express GVs or externally modifying phase‐changing agents as stable cavitation nuclei, the transformation of liquid nanodroplets into gas microbubbles via acoustic droplet vaporization provides additional nuclei for inertial cavitation. This synergism not only enhances localized energy deposition but also minimizes off‐target thermal damage to intervening tissues by reducing the necessary external acoustic dose [14].

SDT utilizes ultrasound‐activated sonosensitizers to generate ROS, primarily singlet oxygen (^1^O_2_), to induce oxidative damage in tumor cells [80]. The fundamental efficiency of SDT is governed by the availability of acoustic energy and local oxygen concentrations. However, the efficacy of conventional SDT is often hindered by the hypoxic TME, which severely constrains the oxygen‐dependent ROS generation [81]. To overcome these physical‐chemical bottlenecks, URBs can be engineered as biological modulators of the TME. Mechanistically, bacteria can be programmed to perform enzymatic catalysis or photosynthesis to alleviate hypoxia, thereby providing a sustained substrate supply for SDT. For example, the expression of catalase within engineered bacteria enables the localized decomposition of endogenous H_2_O_2_ into O_2_, while the use of photosynthetic strains (e.g., cyanobacteria) can generate O_2_ in situ upon light stimulation. By elevating the local oxygen partial pressure, these bacterial systems facilitate the transition of sonosensitizers from an excited state to a triplet state more effectively, resulting in amplified ROS production under ultrasound irradiation compared to standalone acoustic sensitizers [31, 82].

#### 3.3.4 Bacteria‐Assisted Ultrasound Imaging and Theranostics

Ultrasound contrast imaging, as a real‐time dynamic monitoring technology, operates on the core principle of enhancing echo signals through the scattering of ultrasound waves by microbubbles [83]. URBs establish a visualizable therapeutic feedback mechanism. This strategy utilizes changes in acoustic signals to characterize the dynamic process of drug release in real time. Specifically, following the targeted colonization of tumors by bacteria incorporating specific acoustic‐responsive elements, these elements undergo a phase transition upon ultrasound excitation. This process not only initiates the therapy but also generates microbubbles that instantaneously enhance the ultrasound echo signal. Given that microbubble generation is synchronous with carrier rupture, this intense echo signal serves as an indicator of drug release. By monitoring the signal‐enhanced regions on the screen, one can directly determine whether the drug has been successfully released at the target site, thereby determining the optimal timing for HIFU treatment [35, 84].

Furthermore, to further enhance the precision of image guidance, multimodal imaging strategies have been introduced. Wang et al. constructed an URB that achieved trimodal imaging—fluorescence, MRI, and US. This multi‐dimensional visual information enables complementary verification and provides real‐time feedback on the status of the tumor microenvironment from different levels, thereby guiding the real‐time optimization of ultrasound treatment parameters [35].

### 3.4 Advantages of Ultrasound‐Responsive Bacteria

Ultrasound‐responsive bacteriotherapy is an emerging therapeutic strategy, which can not only utilize the physical properties of ultrasound to enhance the effect of bacteriotherapy, but also utilize the carrier function of bacteria to enhance the ultrasound therapy, the advantages of which are mainly reflected in the following aspects.

First, the endogenous lactic acid accumulated during bacterial therapy exacerbates the tumor immunosuppression, which contributes to the immune escape and the failure of immune surveillance. ROS generated by SDT modulates bacterial amplification and inhibits immunosuppression‐associated lactate production. This combination enhances the immune response to antitumor and the activation of anti‐metastatic immunological memory, leading to the growth of microbiota, increased tumor lysis, and accurate control of lactate metabolism.

Second, bacteria have the potential to serve as targeting vectors and boosting the efficacy of the ultrasound therapy due to their unique bio‐properties is possible. Bacteria can colonize the TME and release ultrasound potentiators to enhance the targeting of the therapy. One such example is the genetically engineered bacteria, which can be loaded with GVs and PFH that can serve as cavitation nuclei when subjected to HIFU irradiation, which boosts the cavitation effect and thereby the efficiency of energy deposition to a large extent. In this way, more energy is supplied to deep tumors and less to normal tissues, which are destroyed. Metabolites of bacteria promote oxygenation in tumors. By overexpressing catalase, bacteria can decompose H_2_O_2_ into O_2_ in hypoxic regions of tumors, thereby increasing local oxygen partial pressure. This boosts ROS production. Enhancement of the tumor area with the use of the ultrasonic booster enables the tumor cells to strike the tumor area with precision, causing minimal damage to tissue. The approach enhances the effectiveness of therapeutic interventions and minimizes adverse reactions and pain in patients.

Moreover, URB therapies enable the time‐ and space‐controlled release of drugs or genes, avoiding the systemic toxicity and side effects associated with conventional bacterial therapies. The ultrasound activates drug release systems that compare therapeutic medications to be released at the right time and location, enhancing the efficacy and reducing side effects. Inertial cavitation can be produced by ultrasound, which destroys drug carriers to deliver drugs locally. Specificity of gene therapy is achieved by using gene carriers and ultrasound to deliver genes at a certain time and location. The enhanced expression of temperature‐sensitive genes has allowed the expression of genes during ultrasonic irradiation, and the temperature‐sensitive transcriptional deterrent proteins can effectively silence particular genes to allow the manipulation of gene expression.

Lastly, the therapy enables real‐time monitoring and customisation of the treatment through altering the treatment plan. It is possible to observe non‐invasively that tumor cells make use of genetically coded acoustic reporter genes to monitor medication release and tumor progression. Such real‐time monitoring increases treatment safety and reduces patient discomfort.

## 4 Application of Ultrasound‐Responsive Bacteria in Cancer Therapy

URBs provides an avenue for multimodal synergistic cancer therapy. This model not only achieves synergistic treatment of ultrasound and bacteria but also enables spatiotemporal control, minimizing damage to normal tissues [85]. It also builds on this foundation by combining chemotherapeutic drugs [14], genetic engineering [86], acoustic sensitizers [39], contrast microbubbles, [35] and radiotherapy [30] for drug release, gene expression, immune activation, and real‐time imaging. When applying the above treatments alone, the treatments are unable to quickly localize to the tumor cells to play a role, which poses a greater threat to normal tissues. This combined mode also makes up for the defect that URBs cannot significantly inhibit tumor growth and improves the efficiency of tumor treatment. In conclusion, URB‐based tumor therapy has received extensive attention and rapid development in the past five years (Table 1). Therefore, a comprehensive review is urgently needed to summarize the advances in this field and guide future developments, as well as to present the advantages and limitations of multimodal synergistic therapy with URBs.

**TABLE 1:** Recent cancer therapeutic strategies based on ultrasound‐responsive bacterial biotherapy.

Bacterial strain	US‐responsive materials	US parameters	Combination method	Combination therapies	Tumor type	Refs.
*E. coli* MG1655	Temperature‐actuated genetic switch	4.93 MPa,960 Hz,30 min	Plasmid construction	INF‐γ under heat irradiation.	4T1, H22‐Luc	[15]
*E. coli* Nissle1917	HPNDs	1 MHz, 1.4 W/cm^2^, DC 50%, 10 min	Carbodiimide chemistry	Bacteriotherapy and SDT	TNBC	[39]
*E. coli* DH5α	IR780	1 MHz, 1 W/cm^2^, 2 min	Membrane infusion and nanoparticle coating	Immunomodulatory therapy and SDT	4T1	[24]
*E. coli* BL21 (DE3)	SPNPs3	1.5 W/cm^2^	TK bond	Immunotherapy and SDT	4T1	[18]
*E. coli* BL21(AI)	GVs‐*E. coli* PTX‐CLs	0.94 MHz, diameter 220 mm, focal length 170 mm, 120 W for 3 s	Genetic modification, electrostatic adsorption	Chemo‐synergistic therapy and HIFU	4T1	[14]
*E. coli* Nissle1917	TcI42	0.67 MHz, 0.6‐0.7 MPa, DC 50%, 1h	Genetic modification	Immune checkpoint inhibitors and FUS	A20	[16]
VNP20009	TlpA39	0.5 W/cm^2^, pulse of 1s on, 1s off	Sono‐activatable integrated gene circuit (SINGER)	Poptotic proteins and immune checkpoint inhibitors	B16F10, CT26, A20, H22	[17]
*E. coli* BL21(AI)	GVs‐*E. coli*	Diameter 100–300 mm, focal length 100–250 mm, 0.5–2 MHz	Genetic modification	Bacteriotherapy and HIFU	MDA‐MB‐231	[102]
Cyanobacteria	Mn_1.4_WO_x_	1.0 MHz, DC 50%	Electrostatic adsorption	SDT	4T1	[82]
*E. coli* BL21	PCN NPs	1 MH, 1.5 W/cm^2^	Electrostatic adsorption	Bacteriotherapy and SDT	CT26	[31]
*E. coli*	TiO_1+x_	1 MH, 1.5 W/cm^2^	Amide linkage and hydrothermal method	SDT and immunotherapy	4T1	[103]
*E. coli* MG1655	DOX‐PFP‐PLGA	1 W/cm^2^, 1 MHz, 0–10 min	Amide bond	Diagnosis and treatment	4T1	[34]
*E. coli* BL21	HPP	1 W/cm^2^, 1 MHz, DC 50%, 10 min	Carbodiimide chemistry	Bacteriotherapy and SDT	4T1	[84]
*B. longum*	CL‐ICG‐PFH‐NPs	Focal length 100–250 mm, diameter 100–300 mm, 0.5–2 MHz, 150 W	Electrostatic adsorption	Bacteriotherapy and HIFU	MDA‐MB‐231	[104]
*E. coli*	GVs‐*E. coli* IR780‐PFH‐AQ4N	Focal length 145 mm, diameter 220 mm, 1 MHz, 120–150 W	Genetic modification, electrostatic adsorption	Chemotherapy and HIFU	4T1	[105]
*E. coli*	GVs‐*E. coli*	0.72–1.74 MPa, 86.8 Hz	Genetic modification	Bacteriotherapy	Tumors in general	[29]
*E. coli* BL21(AI)	GVs‐*E. coli* PEI‐PLGA/EPI/PFH@Fe_3_O_4_	150 W, DC 50% (6s) or DC 10% (30s), 1 MHz, focal length 145 mm, diameter 220 mm	Genetic modification, electrostatic adsorption	Chemotherapy and FUAS	4T1	[35]
*B. bifidum*	PEI‐PLGA‐NaHCO_3_‐NPs	1 MHz, DC 10%, 120 W, 3 s	Electrostatic adsorption	Bacteriotherapy and HIFU	4T1	[37]
*B. bifidum*	CPNs	150 W, 2s	Electrostatic adsorption	Bacteriotherapy and HIFU	MDA‐MB‐231	[36]
*B. bifidum*	CL‐PFH‐DOX‐NPs	1 MHz, DC 10%, 120 W, 3 s	Electrostatic adsorption	Chemotherapy and PFUS	4T1	[22]
*B. longum*	PFH/BL‐NPs	Focal length 220 mm, diameter 145 mm, 0.94 MHz, 250 W, 5 s	Cross‐linked	Bacteriotherapy and HIFU	VX2	[40]
*B. longum*	PFH/PLGA NPs	150 W, 3 s	Carbodiimide method	Bacteriotherapy and HIFU	MDA‐MB‐231	[38]
*S. typhimurium*	LTSL	DC 35%, 5 Hz, 6 W	Biotin‐streptavidin chemistry	Chemotherapy and HIFU	C26	[12]
*E. coli* MG1655	GVs‐*E. coli* pBV220	1 MHz, DC 66.7%, 4.93 MPa, 25 min	Cis‐aconitic anhydride	Chemotherapy and immunotherapy	4T1	[23]
*E. coli* MG1655	pBV220	1 MHz, 0.4 W/cm^2^, DC 80%, 30 min‐2 h	Plasmid construction	Bacteriotherapy and radiotherapy	4T1	[30]
*E. coli* MG1655	PpIX	1 MHz, 0.75 W/cm^2^, 10 min	Plasmid construction	SDT and immunotherapy	4T1	[106]
*E. coli* MG1655	pBV220‐CAT	1.6 W/cm^2^, 1 MHz, 3s ON / 7s OFF, 30 min	Plasmid construction	Bacteriotherapy and radiotherapy	4T1	[107]
*E. coli* Nissle1917	pBV220	4 W, 6 min	Plasmid construction	Oral vaccines and immunotherapy	CT26	[74]

### 4.1 Drug Release

Spatiotemporal precisely controlled drug delivery is an important application of ultrasound‐responsive bacterial therapy in tumor treatment [87]. Unlike normal tissue physiology, tumor cells have capillaries that are prone to leakage [88], which poses a challenge for drug penetration. As a result, overdosing or administering prodrugs in an overactive form is usually preferred in drug therapy for tumors [89]. In contrast, URBs exhibit an intrinsic tropism for TME, which facilitates sustained intratumoral drug accumulation [15]. Moreover, their ultrasound responsiveness enables precise spatiotemporal control over drug release or activation via non‐invasive external modulation. This dual mechanism not only enhances therapeutic efficacy but also significantly mitigates the systemic toxicity associated with conventional chemotherapy [16], rendering URBs an ideal vehicle for targeted drug delivery.

**FIGURE 6:**
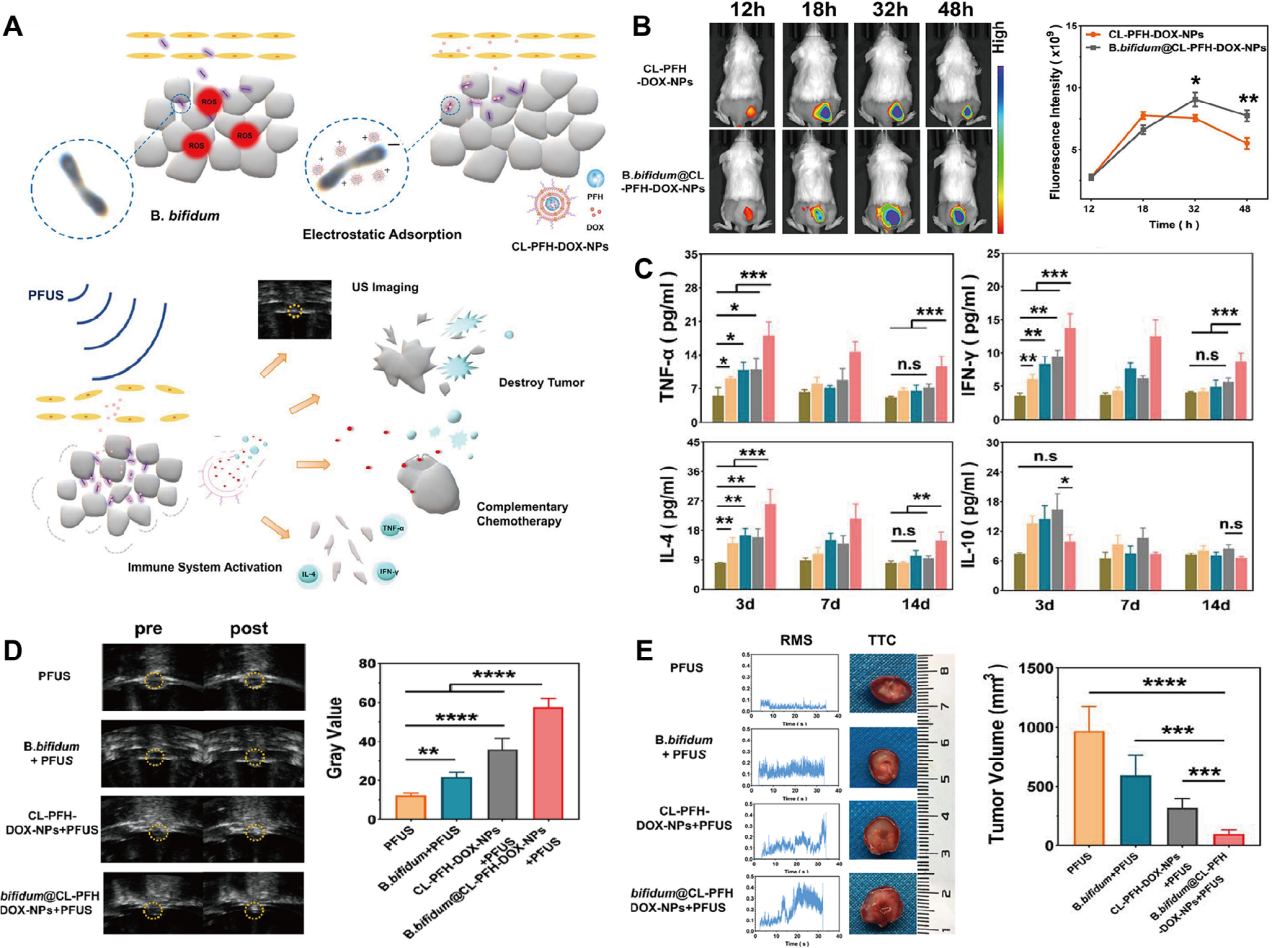
(A) Illustration of the synthetic process and combined antitumor action of B.bifidum@CL‐PFH‐DOX‐NPs. (B) The spatiotemporal distribution and fluorescence intensity quantification of CL‐PFH‐DOX‐NPs within tumors at indicated times. (C) Comparative analysis of grayscale alterations and values in different groups before and after PFUS exposure. (D) Dynamic changes in serum levels of TNF‐α, IFN‐γ, IL‐4, and IL‐10 among the experimental groups. (E) Assessment of tumor necrotic areas, RMS values during PFUS application, and representative TTC staining following treatment. Reproduced with permission [22]. Copyright 2023, RSC Publishing.

A variety of chemotherapeutic drugs, including DOX, cisplatin, etoposide, ifosfamide, and AQ4N, have been successfully loaded onto URBs for tumor‐targeted delivery [34, 90]. Ou et al. constructed an URB utilizing *B. longum* and cationic lipid nanoparticles co‐laden with PFH and DOX, which enabled precise delivery of antitumor drugs (Figure 6A) [22]. Figure 6B shows that *B. longum* mediated a robust increase in DOX accumulation within tumors, demonstrating its role in promoting drug enrichment. The ultrasound‐responsive nano‐bio complex elicited a robust anti‐tumor immune response, thereby synergistically potentiating the overall therapeutic efficacy (Figure 6C). Meanwhile, CL‐PFH‐DOX‐NPs significantly enhanced the ultrasound imaging effect under the action of pulsed‐focused ultrasound (PFUS) (Figure 6D), and effectively solved the problems of insignificant sonication effect, prolonged treatment time, and tumor residue remaining in unirradiated area due to low duty cycle of PFUS for solid tumor therapy (Figure 6E).

**FIGURE 7:**
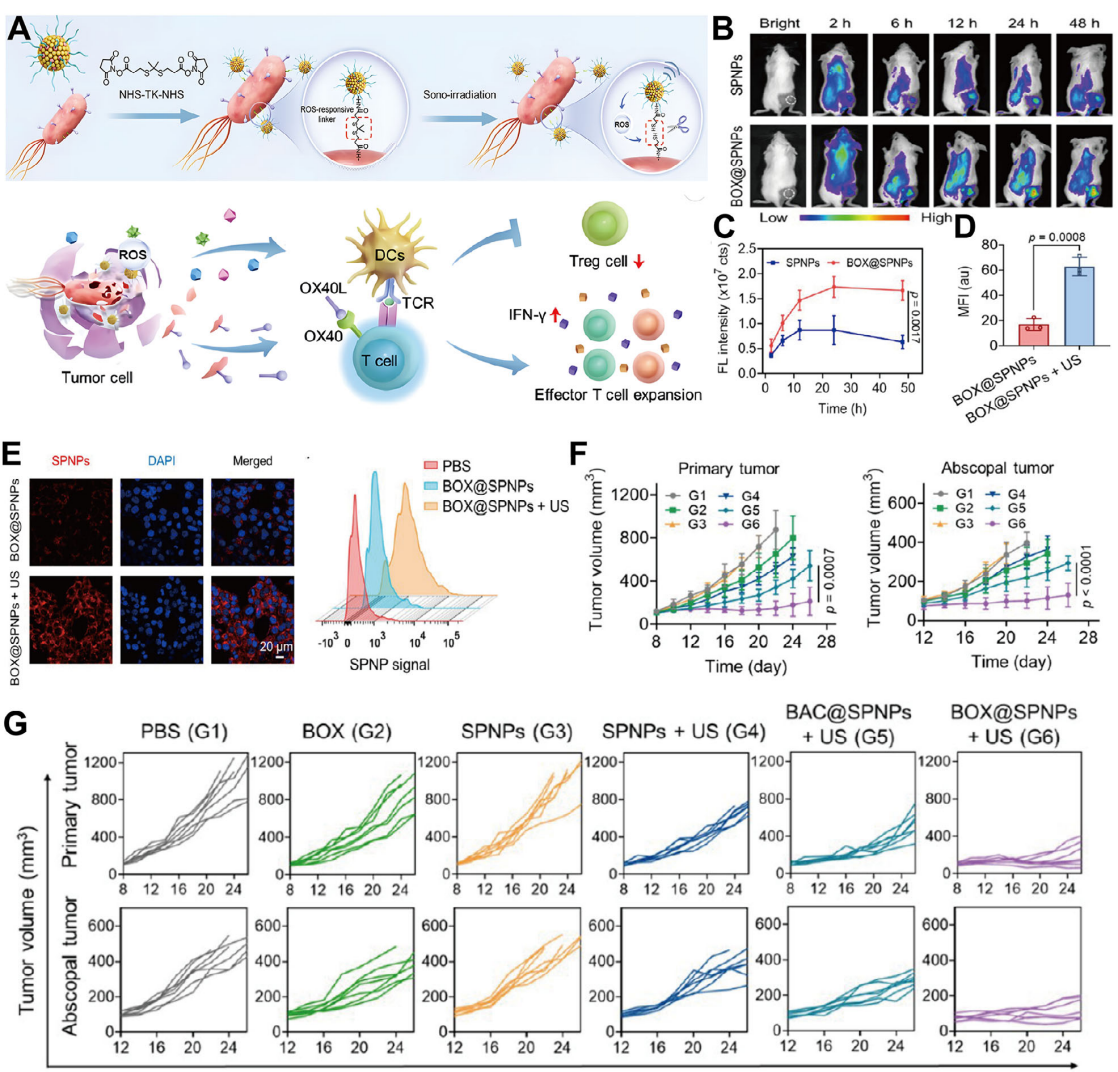
(A) Schematic of the ultrasound‐powered intelligent biohybrid. (B) Spatiotemporal distribution in 4T1 tumor‐bearing mice after intravenous injection of SPNPs or BOX@SPNPs, monitored by in vivo NIR fluorescence imaging. (C) Quantitative analysis of the corresponding fluorescence intensity at the tumor site. (D,E) Bottom 4T1 cells were incubated with BOX@SPNPs in the upper chamber and processed with or without US irradiation for CLSM images, quantification of NP fluorescence signal, and flow cytometry analysis of NP fluorescence signal. (F,G) Growth trends and average tumor volumes of primary and abscopal tumors after specified treatments. Reproduced with permission [18]. Copyright 2025, American Chemical Society.

URB can also be utilized to deliver drugs using acoustic sensitizers. Ultrasound activates the acoustic sensitizer to generate ROS, which further breaks the ROS‐responsive chemical bond to release the drug. Based on the above design concept, Liang et al. constructed an innovative bacterial‐mediated acoustic sensitization immunotherapy system (BOX@SPNPs). Bacteria and sensitizer nanoparticles were connected by ROS‐responsive TK bonds to realize a cascade response triggered by ultrasound and enhance the therapeutic effect (Figure 7A) [18]. In vitro experiments showed that under ultrasound excitation, the released SPNPs were able to get closer to the tumor cells, thereby generating higher concentrations of ROS locally and enhancing the therapeutic effect of acoustic kinetic therapy (Figures 7D,E). In vivo studies confirmed that BOX@SPNPs robustly colonized and persisted within tumor tissue (Figures 7B,C). significantly inhibited primary tumor growth, and exerted a distal tumor suppressive effect by activating systemic immune responses (Figures 7F,G). In addition to this, precise drug release and immune activation can also be achieved by combining FUS and URB carrying LTSL. Interestingly, antitumor drug‐mediated lysis of tumor cells could lead to the release of nutrients that promote bacterial metabolism and growth, thus improving bacterial therapy.

### 4.2 Gene Expression

Controlling the spatiotemporal expression of inserted genes at the site of solid tumors has been a challenge. Conventional chemical inducers lack specificity [91], biological methods are difficult to achieve precise spatiotemporal control [92], while physical methods such as photostimulation are limited due to poor tissue penetration [93], and ionizing radiation risks damaging host immune cells and engineered microbial cells [94]. Ultrasound activation of bacteria‐carried gene expression systems solves this challenge. FUS serves as an external energy delivery platform for non‐invasive ablation of solid tumors. Genetically engineered bacteria carry synthetic gene circuits with temperature‐sensitive promoters. When the bacteria localize to the tumor tissue, FUS activates the promoter, causing the bacteria to persistently express anti‐tumor proteins and secrete immune factors, such as interleukins and TNF‐α [85]. These proteins and factors can either directly kill tumor cells or enhance the activity of immune cells activated at the tumor site or recruit more immune cells, thus significantly improving the efficacy [15, 23]. For example, Chen et al. constructed an ultrasound‐responsive gene expression system for precise control of gene expression. The URB was prepared by inserting the gene encoding IFN‐γ into the temperature‐sensitive plasmid vector pBV220 and transforming it into *E. coli* MG1655. pBV220 possesses a gene switch constructed by the temperature‐sensitive PL/PR phage promoter and the cI857 inhibitory protein, and under the FUS irradiation, the URB utilizes the thermal effect to activate this switch, which triggers the expression of IFN‐γ expression.

**FIGURE 8:**
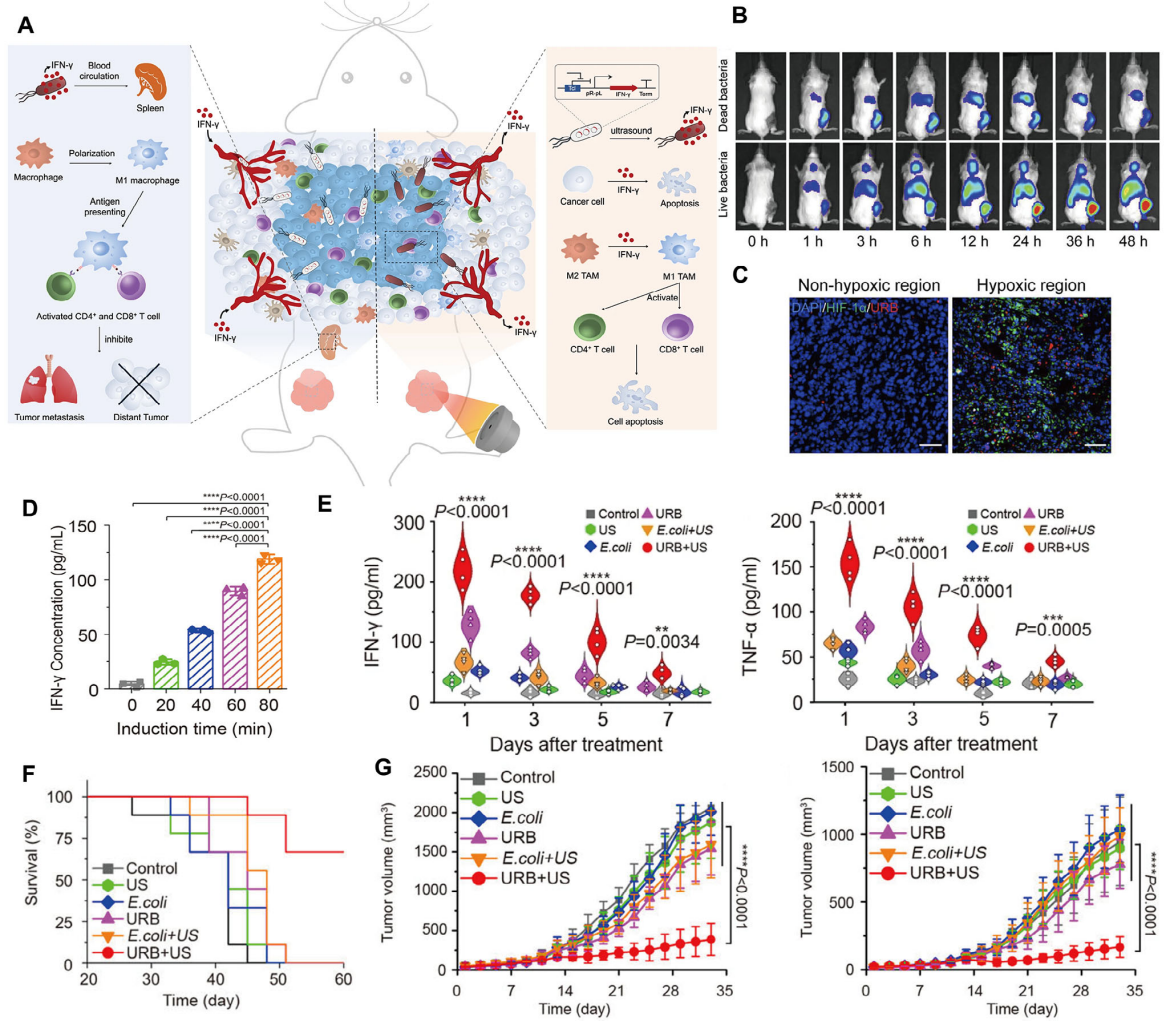
(A) Schematic of URB in controlling IFN‐γ expression by focused ultrasound and their mechanisms for cancer immunotherapy. (B) In vivo fluorescence images of tumor‐bearing mice injected with 1 × 10^7^ CFU of either live or heat‐inactivated DiR‐URB, captured at various time points. (C) Immunofluorescence staining of tumor sections from the peritumoral (left) and central (right) regions. (D) Analysis of necrotic volume, the RMS during PFUS, and TTC staining of tumors post‐PFUS. (E) Intratumoral levels of TNF‐α and IL‐1β on days 1, 3, 5, and 7 after treatment. (F) Survival curves for the different groups. (G) Growth of primary (left) and distant (right) tumors in mice receiving various treatments. Reproduced with permission [15]. Copyright 2022, Springer Nature.

As can be seen in Figure 8A, this system enhances the infiltration of M1 macrophages into tumors, thereby promoting the secretion of pro‐inflammatory cytokines and leading to the activation of antigen‐specific T cells [15]. Figures 8B,C demonstrated that URBs were able to specifically accumulate in tumor and penetrate deep into tumor. Triggered by ultrasound, the local temperature was increased to 45°C to achieve precise spatiotemporal expression of IFN‐γ in deep tissues, and the expression increased with the prolongation of ultrasound irradiation time (Figure 8D). It can effectively induce a significant increase in the levels of immune factors such as TNF‐α and IL‐1β, and activate the anti‐tumor immune response (Figure 8E). Figure 8F shows that the treatment group exhibited significantly prolonged survival time compared with the control group, providing an innovative strategy for the spatiotemporal control of gene expression. Not only that, this system can also activate immune cells in the spleen by activating the immune cells in the spleen, so it demonstrated significant anti‐tumor effects, including the inhibition of primary tumors, distant tumor growth and metastasis (Figure 8G).

**FIGURE 9:**
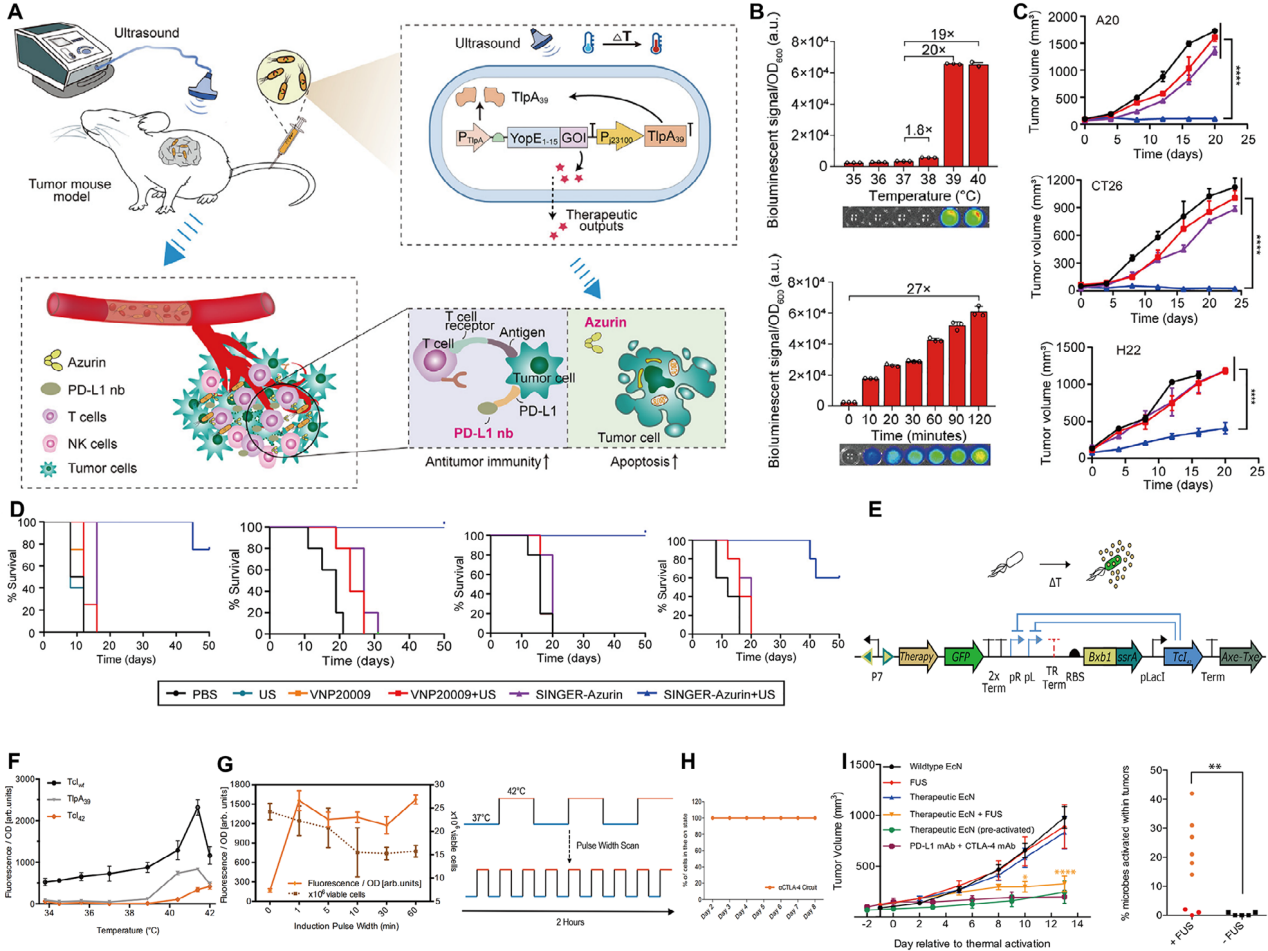
(A) Schematic of the US‐triggered, bacteria‐based therapeutic strategy in xenograft mouse models. (B) Transgene expression levels in engineered bacteria profiled under different thermal induction conditions and ultrasound exposure times at 39°C. (C,D) Tumor volume measurements and Kaplan–Meier survival curves of mice bearing B16F10, CT26, A20, or H22 xenografts. Reproduced with permission [17]. Copyright 2024, Elsevier Inc. (E) Schematic of the genetic circuit used to establish a temperature‐responsive state switch. (F) EcN cells transformed with reporter gene circuits were incubated at different temperatures (33°C to 42°C) for 1 h and the optical density (OD600)‐normalized fluorescence was measured. (G) Stability of gene expression in thermally induced circuits. (H) Schematic of the pulsed heating protocol designed to simultaneously optimize thermal induction and preserve cell viability; orthogonal fluorescence of the TcI42 circuit is shown as a function of pulse width. (I) Left: Tumor growth kinetics over two weeks in mice receiving wild‐type EcN, therapeutic microbes without FUS, therapeutic microbes plus FUS, or FUS alone. Right: Percentage of activated therapeutic EcN retrieved from FUS‐treated (*n* = 9) vs. non‐FUS‐treated (*n* = 5) tumors after two weeks. Reproduced with permission [16]. Copyright 2022, Springer Nature.

Another gene regulatory circuit for URB is the SINGER (sono‐activatable integrated gene circuit) system, which is built upon the thermally responsive transcription factor TlpA39. Like the above system, it uses ultrasound‐triggered thermal stimulation to activate the expression of target proteins. Gao et al. developed the SINGER system to enable precise expression and secretion of therapeutic proteins Azurin and PD‐L1 nb, thereby directly triggering tumor cell apoptosis and potentiating anti‐tumor immune responses. (Figure 9A) [17]. The SINGER system can be activated at 39°C, which is highly sensitive and further avoids thermal damage to healthy tissues (Figure 9B). The SINGER system can significantly inhibit tumor growth in superficial tumors, such as B16F10 melanoma, CT26 colon cancer, A20 B‐cell lymphoma, and H22 hepatocellular carcinoma, and prolonged the survival of mice, with no obvious side effects (Figures 9C,D). Since effective tumor suppression demands prolonged intervention over weeks, Mohamad et al. optimized bacterial switch circuits to sustain long‐term therapeutic response following a single transient thermal activation. They screened a library of gene circuit variants to identify constructs exhibiting the lowest basal activity and the highest thermostimulation‐induced activity. The final choice was to construct a gene circuit utilizing the temperature‐sensitive transcription factor TcI42 and the integrase Bxb1 to achieve the release of long‐acting immune checkpoint inhibitors (CTLA‐4 and PD‐L1 antibodies) after transient thermal stimulation (Figure 9E) [16]. At the permissive temperature of 42°C, TcI42 showed stronger induction and lower basal activity than other transcriptional inhibitors (Figure 9F). And a pulsed heating scheme was used to ensure robust activation of URBs while minimizing cellular damage (Figure 9G). Figure 9H shows that the optimization of this therapeutic gene circuit does not affect bacterial activity.

### 4.3 Immune Activation

Immunotherapy centers on the use of the autoimmune system to fight cancer, with high specificity and long‐lasting efficacy. Immunotherapy has made important clinical progress in recent decades. However, most of them are only used for malignant hematological diseases [95]. Solid tumors have a complex microenvironment and immunosuppressive properties, direct infusion of immune factors may not be effectively enriched at the site of solid tumors, and hypoxia and immunosuppressive TME also inhibit the function of immune cells, which leads to limited therapeutic efficacy [96, 97]. Some patients clinically developed an autoimmune response [98].

**FIGURE 10:**
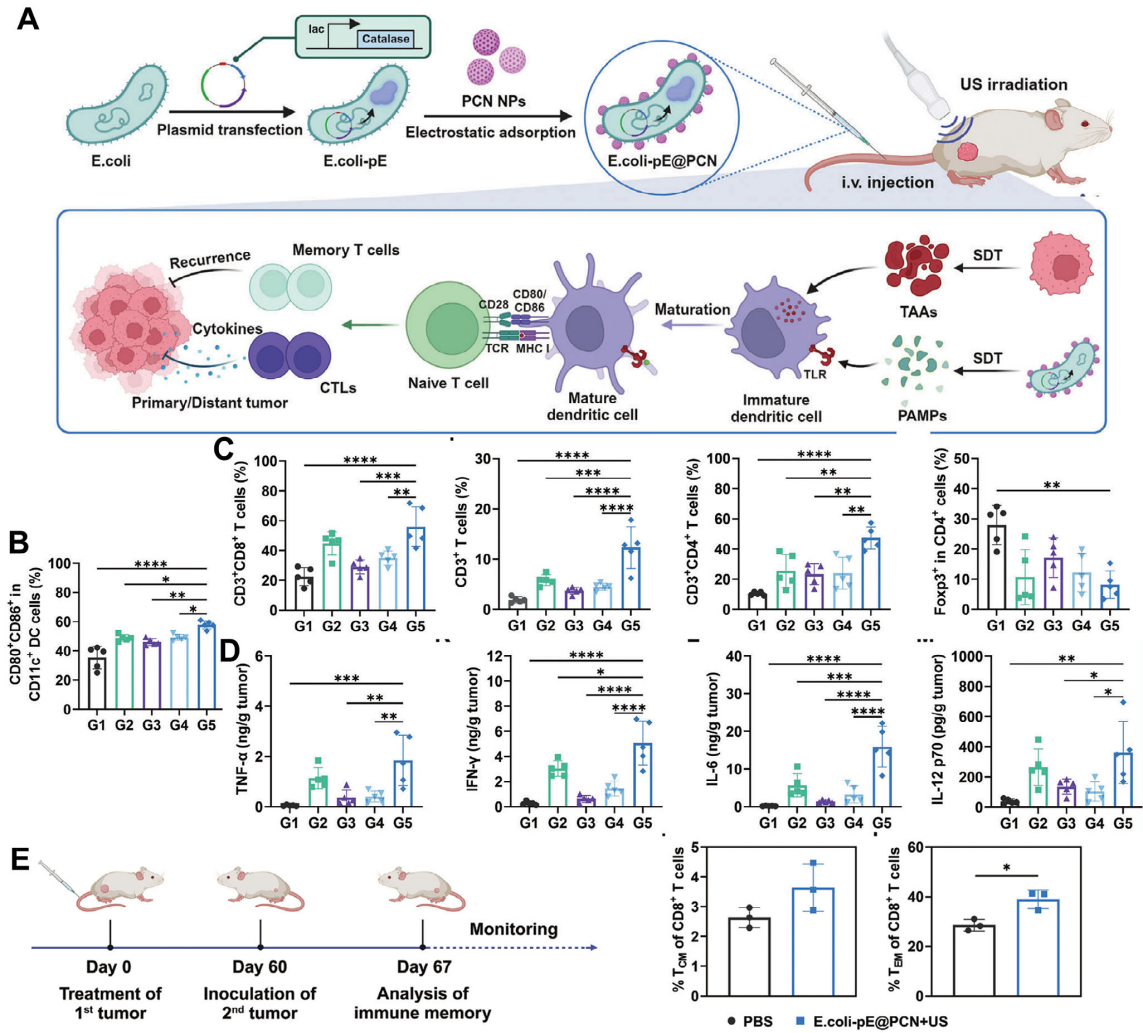
(A) Schematic of the synthesis process and synergistic antitumor action of E. coli‐pE@PCN. (B) Flow cytometric analysis of intra tumoral DCs maturation in CT26 tumor‐bearing mice. (C) Proportions of CD3^+^CD8^+^ T cells, total CD3^+^ T cells, CD3^+^CD4^+^ T cells, and CD4^+^Foxp3^+^ Tregs within tumors across treatment groups. (D) Pro‐inflammatory cytokines in tumors from differently treated mice. (E) Experimental timeline and the percentage of CD8^+^ TEM T cells in the plasma of healthy and cured mice on day 60. Reproduced with permission [31]. Copyright 2024 Wiley‐VCH GmbH.

On the contrary, this TME is very favorable for the growth of URBs, which possess tumor‐infiltrating properties. Therefore, URBs can be used as a local immune activator for precise immune activation. First, URBs have a natural immune activation potential, especially in the TME, and are able to stimulate immune responses through their surface antigens and secreted immunoreactive factors [99]. For example, flagella on the surface of Salmonella can bind to TLRs and increase the immunogenicity of antigens [100]. Second, URB types carrying acoustic sensitizers can mediate SDT and directly activate the immune response [101]. Wang et al. developed a novel bacterial biohybrid system (*E. coli*‐pE@PCN) for enhanced and targeted acoustic‐dynamic immunotherapy by combining genetically‐modified *E. coli* expressing catalase with the acoustic sensitizer nanoparticle PCN (Figure 10A) [31]. It was demonstrated that SDT treatment induced the maturation of approximately 58% of DCs, which are key antigen‐presenting cells for activating immune responses (Figure 10B). Not only that, the number of total T cells, cytotoxic T lymphocytes, and CD4^+^ helper T cells was also increased, and the number of immunosuppressive cells Tregs was decreased (Figure 10C). The treatment also induced elevated levels of the pro‐inflammatory cytokines, activating a strong anti‐tumor immune response (Figure 10D).

In addition, SDT could also induce the generation of immune memory, which enabled the organism to rapidly activate an immune response when encountering tumor antigens again, preventing tumor recurrence (Figure 10E). Third, URBs can also remodel the immunosuppressive microenvironment of solid tumors by integrating immune‐associated factors into the genetic circuit of engineered bacteria. URBs expressing immune checkpoint inhibitors (αCTLA‐4 and αPD‐L1 nanoantibodies), and immune factors (IFN‐γ) have now been successfully developed, all of which can successfully activate the body's antitumor immune response. The gene circuit optimized by Mohamad et al. can remain active for at least two weeks when its thermal switch is triggered in the target tumor, continuously secreting immune checkpoints at the tumor site inhibitors, avoiding therapeutic interruptions due to drug metabolism or clearance (Figure 9I) [16]. In contrast, conventional immune checkpoint inhibitors have a short in vivo half‐life, often necessitating repeated dosing to sustain efficacy.

## 5 Summary and Perspectives

Given the remarkable potential demonstrated by the combination of ultrasound technology and engineered bacteria in enhancing tumor therapy, numerous sophisticated strategies have emerged in recent years, highlighting the necessity for a systematic review in this field. This review firstly introduces design strategies for URBs, followed by separate discussions on the roles of ultrasound and bacteria in tumor therapy. It then focuses on exploring the interaction mechanisms and synergistic effects between engineered bacteria and ultrasound. Finally, it systematically reviews current application directions for URB systems. Despite the widespread attention garnered by the integration of engineered bacteria and ultrasound technology, several key scientific questions remain to be resolved.

### 5.1 Ultrasound‐Responsive Bacteria: Challenges and Opportunities

Typically, many drugs fail in clinical trials due to discrepancies between preclinical models and human patients, resulting in insufficient efficacy and excessive toxicity. Bacterial therapies face this same challenge, which must be overcome to achieve the milestone of clinical translation for engineered bacterial therapies. First, an ideal drug delivery system remains to be developed. The reliability of currently used biomaterials is still questionable due to insufficient data and evidence supporting their long‐term biosafety.

Second, the complex structure and acoustic properties of human tissues directly limit ultrasound propagation efficiency and effective penetration depth, thereby constraining therapeutic drug penetration. Future research should focus on enhancing the targeting and penetration capabilities of bacterial systems under ultrasound‐responsive conditions. To successfully advance ultrasound‐responsive bacterial systems toward clinical therapeutic applications, it is essential to thoroughly evaluate active vs. passive drug targeting strategies, tumor microenvironment characteristics, and the overall performance of drug delivery systems.

Previous studies demonstrate that engineered bacteria can accumulate and proliferate specifically in tumor regions. However, a critical question remains: Can engineered bacteria be internalized by cancer cells? If internalized, they would be degraded within lysosomes, thereby losing their anticancer activity. Therefore, monitoring cancer cell uptake of bacteria and measuring uptake efficiency are crucial for customizing engineered bacteria for cancer therapy. Current bacterial visualization relies on multiple imaging modalities—such as fluorescence imaging (FI), photoacoustic imaging (PAI), radionuclide imaging (RNI), and magnetic resonance imaging (MRI)—which detect signals from exogenous contrast agents or endogenous reporter gene expression. This study establishes an ultrasound‐controlled real‐time imaging‐guided bacterial therapy platform, paving the way for precision tumor treatment. This technology enhances therapeutic molecule expression and boosts immunotherapy efficacy. However, challenges remain, such as the inability to directly quantify protein levels from imaging signals and limitations in laser penetration. Further research and improvements are needed in the future.

### 5.2 Perspectives of Ultrasound‐Responsive Bacteria in Cancer Therapy

URBs demonstrate significant potential in cancer therapy, with key developments centring on constructing more intelligent bacterial carrier systems to achieve spatio‐temporal controllability during treatment. Researchers may develop novel bacterial carriers exhibiting both high ultrasonic responsiveness and microenvironment adaptability. Such systems hold promise for dynamically regulating gene expression under specific ultrasonic signals, enabling targeted drug delivery, immune modulation, and other anti‐tumour functions. This approach holds promise for overcoming limitations of conventional therapies and pioneering novel models of personalised treatment.

Furthermore, interdisciplinary integration is pivotal to advancing this field. By integrating synthetic biology, nanotechnology, and acoustic feedback mechanisms, real‐time monitoring and optimisation of bacterial distribution, activity, and therapeutic responses can be achieved, thereby enhancing treatment safety and controllability. Concurrently, efforts should focus on translating fundamental research into clinical applications, promoting the systematisation and standardisation of this strategy. Specifically, future research should prioritise the following directions: First, developing intelligent bacterial carriers to optimise their targeting, colonisation capacity, and release efficiency; Second, establishing more refined ultrasound control strategies to enhance the spatial precision and penetration capability of drug delivery; Thirdly, strengthening interdisciplinary collaboration to advance preclinical validation and translational research.

In summary, URBs represent a highly promising new direction in cancer therapy. By refining bacterial design and ultrasound control strategies, existing technical bottlenecks can be progressively overcome, accelerating clinical translation and thereby offering cancer patients more effective and safer treatment options.

## Conflicts of Interest

The authors declare no conflicts of interest.

## Data Availability

The authors have nothing to report.
